# Mitochondrial dysfunction and PANoptosis-related signaling in acute central nervous system injuries: mechanistic convergence and therapeutic opportunities

**DOI:** 10.3389/fnmol.2026.1982074

**Published:** 2026-09-30

**Authors:** Hongbo Zhang, Ruoxin Tu, Dawei Zhang, Hao Tian, Hongna Yin

**Affiliations:** 1 Heilongjiang University of Chinese Medicine, Harbin, China; 2 Heilongjiang University of Chinese Medicine Affiliated Second Hospital, Harbin, China

**Keywords:** acute central nervous system injury, mitochondrial dysfunction, mitochondrial quality control, neuroinflammation, PANoptosis, PANoptosome

## Abstract

Acute central nervous system (CNS) injuries comprise a heterogeneous group of disorders caused by vascular or traumatic factors, including ischemic stroke, intracerebral hemorrhage (ICH), traumatic brain injury (TBI), and spinal cord injury (SCI). These injuries initiate secondary cascades that combine metabolic failure, oxidative stress, neuroinflammation, and regulated cell death. Mitochondrial dysfunction is positioned near the center of these processes because impaired bioenergetics, excessive mitochondrial reactive oxygen species (mtROS), abnormal fission and fusion, defective mitophagy, and mitochondrial DNA (mtDNA) release can simultaneously disturb cellular homeostasis and activate inflammatory signaling. PANoptosis is an inflammatory form of regulated cell death that integrates pyroptotic, apoptotic, and necroptotic machinery through PANoptosome complexes. Emerging evidence suggests that mitochondrial stress can create conditions that favor PANoptotic convergence by increasing mtROS and damage-associated molecular patterns (DAMPs), activating DNA-sensing and inflammasome pathways, and sustaining receptor-interacting protein kinase and caspase signaling. This review examines the potential mechanistic links between mitochondrial dysfunction and PANoptosis-related signaling across ischemic stroke, ICH, SCI, and TBI. We summarize changes in mitochondrial bioenergetics, oxidative stress, dynamics, mitophagy, mitochondria-derived vesicles (MDVs), and mitochondrial transfer, and evaluate how these changes intersect with PANoptosis-related signaling. Importantly, we distinguish direct evidence of PANoptosis from studies that only demonstrate coordinated changes in individual death pathways. We also assess therapeutic strategies that target mitochondrial oxidative stress, dynamics, mitophagy, biogenesis, mitochondrial DAMP signaling, and mitochondrial transplantation and transfer. Current findings support mitochondria as a promising upstream intervention point, but complete causal evidence linking mitochondrial damage to defined PANoptosome assembly remains limited in acute CNS injury. Clarifying cell-specific and time-dependent mechanisms will be essential for translating this framework into effective neuroprotective strategies.

## 1 Introduction

Acute central nervous system (CNS) injuries arise from diverse vascular and traumatic insults, yet their secondary responses often converge on metabolic failure, oxidative stress, neuroinflammation, and regulated cell death. Experimental studies in cerebral ischemia–reperfusion and intracerebral hemorrhage (ICH) show that restoring mitochondrial function can reduce oxidative injury, preserve neuronal viability, and improve neurological recovery (Xie et al., 2021; Tashiro et al., 2022). Comparable rescue of mitochondrial redox and bioenergetic control also improves outcomes in spinal cord injury (SCI) and traumatic brain injury (TBI; Zhuang et al., 2023; Guo H. et al., 2024). This convergence places mitochondria at an important upstream position in secondary neural injury. Mitochondria do more than supply adenosine triphosphate (ATP). They also regulate calcium buffering, redox balance, metabolic adaptation, and innate immune signaling (Creus et al., 2026). When damaged, mitochondria produce excess mitochondrial reactive oxygen species (mtROS), lose membrane and respiratory integrity, and release mitochondrial DNA (mtDNA) and other damage-associated molecular patterns (DAMPs; Xiaowei et al., 2023; He et al., 2026). This link becomes especially relevant to PANoptosis, an inflammatory form of regulated cell death that integrates pyroptotic, apoptotic, and necroptotic machinery through multiprotein PANoptosome complexes (Lee et al., 2021). Foundational experiments established that coordinated activation of caspases, receptor-interacting protein kinases (RIPKs), inflammasome components, and gasdermins can produce a death response that cannot be explained by one pathway alone (Zheng et al., 2020; Karki et al., 2021). Recent studies have extended this concept to sterile CNS injury. Z-DNA-binding protein 1 (ZBP1)-dependent PANoptosis has been reported in ischemic neurons and microglia after TBI (Shu et al., 2025; Shuang et al., 2026). A receptor-interacting serine/threonine-protein kinase 1 (RIPK1)-containing PANoptosome has also been demonstrated after experimental subarachnoid hemorrhage (Duan et al., 2025). Mitochondrial dysfunction may provide the upstream environment that favors this convergence. In macrophage models, blocking reverse electron transfer-driven mtROS reduced mtDNA oxidation, PANoptosome formation, and pyroptotic, apoptotic, and necroptotic signaling, providing direct evidence that mitochondrial stress can regulate PANoptosis (Shi et al., 2024). In acute CNS injury, the full causal sequence from mitochondrial damage to defined PANoptosome assembly is still incompletely established (Gao et al., 2026; Pan et al., 2026). This review therefore examines the potential mechanistic convergence between mitochondrial dysfunction and PANoptosis-related signaling across ischemic stroke, ICH, SCI, and TBI, distinguishes direct evidence from mechanistic bridging evidence, and evaluates therapeutic strategies that target mitochondrial stress before multiple death programs become fully engaged.

## 2 Mitochondrial dysfunction and acute CNS injuries

Mitochondria are central regulators of the response to acute CNS injury because they connect ATP production with calcium buffering, redox balance, organelle quality control, and stress signaling (Bai et al., 2024). Experimental ischemia shows that disruption of mitochondrial respiratory complexes, membrane potential, and permeability is closely associated with neuronal injury (Li H. et al., 2025). Hemorrhagic injury likewise damages cristae structure and impairs mitochondrial membrane potential (ΔΨm), while restoration of mitochondrial architecture improves neuronal survival (Deng et al., 2021). After SCI, disturbed mitochondrial quality control contributes to neuronal vulnerability and can be modified by interventions that restore the mitochondrial pool (Yao et al., 2023). TBI also produces persistent mitochondrial dysfunction, and mitochondrial deubiquitination has been directly linked to oxidative damage and secondary brain injury (Wu et al., 2023). These results indicate that different types of acute CNS injury ultimately share relatively consistent mitochondrial abnormality features. Energy metabolism dysfunction, oxidative stress, imbalance between fission and fusion, insufficient clearance of damaged organelles, and aberrant mitochondrial signaling can mutually reinforce one another (Figure 1).

**Figure 1:**
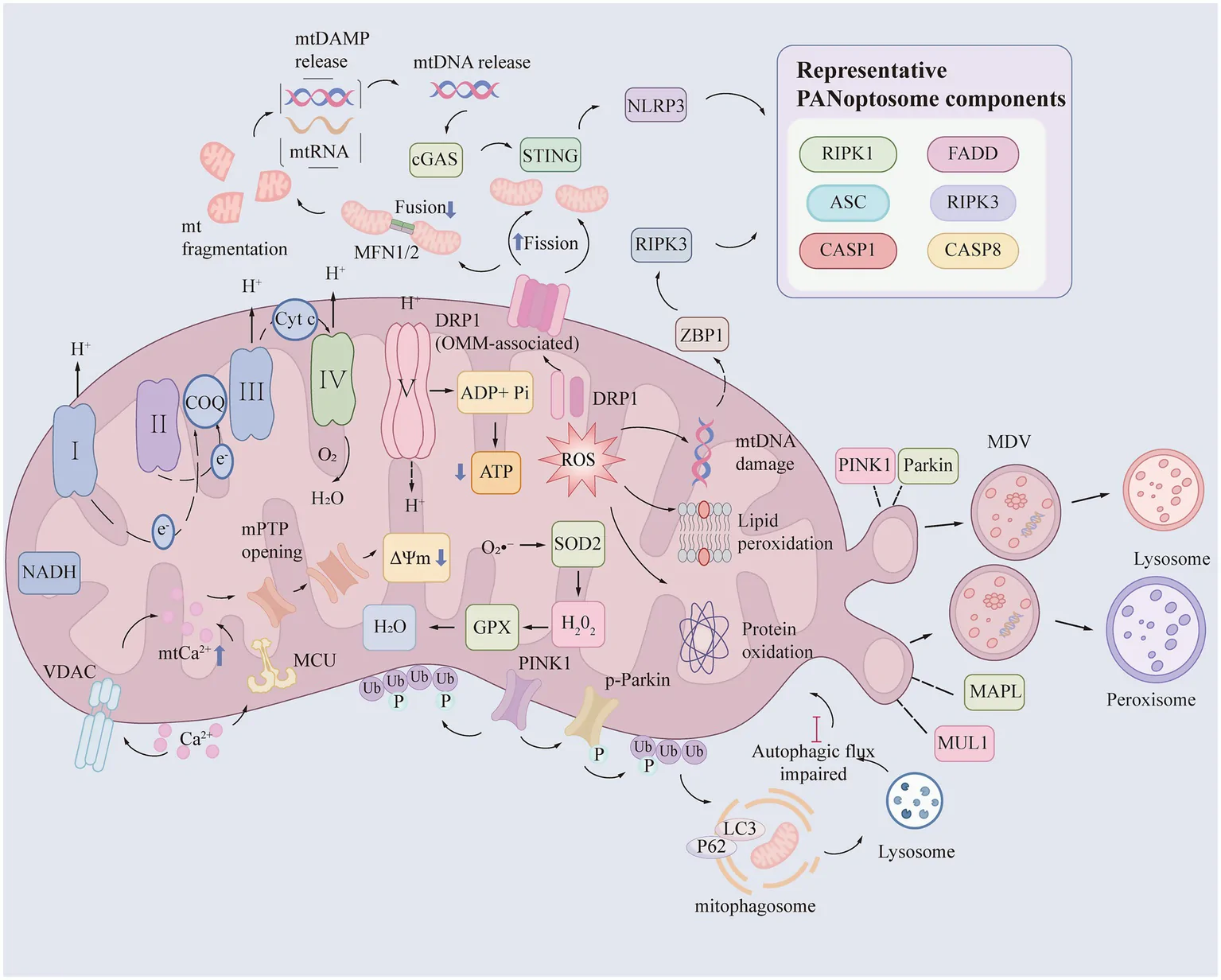
Mitochondrial dysfunction in acute CNS injury. Acute CNS injury can impair the mitochondrial electron transport chain (ETC) and oxidative phosphorylation (OXPHOS), reduce mitochondrial membrane potential (ΔΨm) and ATP production, promote mitochondrial calcium overload and mitochondrial permeability transition pore (mPTP) opening, and increase reactive oxygen species (ROS) generation. Excessive oxidative stress damages mitochondrial DNA (mtDNA), lipids, and proteins. Increased DRP1-dependent mitochondrial fission together with reduced MFN1/2-mediated fusion promotes mitochondrial fragmentation and can facilitate the release of mitochondrial damage-associated molecular patterns (mtDAMPs), including mtDNA, mtRNA, and cardiolipin. Released mtDNA can engage the cGAS–STING pathway and promote NLRP3-associated inflammatory signaling, whereas mitochondrial ROS (mtROS) is associated with ZBP1/RIPK3 signaling. These mitochondrial stress signals may converge on PANoptosome-associated pathways involving representative components such as RIPK1, RIPK3, FADD, CASP8, ASC, and CASP1. In parallel, loss of ΔΨm stabilizes PINK1 and promotes Parkin recruitment, linking mitochondrial ubiquitination to mitophagy, although impaired autophagic flux can limit the clearance of damaged mitochondria. Mitochondria-derived vesicles (MDVs) provide an additional quality-control route for selective mitochondrial cargo trafficking to lysosomes or peroxisomes.

### 2.1 Mitochondrial bioenergetics

Mitochondrial bioenergetics is particularly important in neural tissue because membrane repolarization, synaptic transmission, axonal transport, and repair all require continuous ATP supply (Zhu et al., 2022). In ischemic neurons, preserving mitochondrial iron handling increased ATP content and spare respiratory capacity measured with Seahorse and high-resolution respirometry, providing direct evidence that respiratory competence determines survival under oxygen–glucose deprivation (OGD) and reperfusion (Wang P. et al., 2022). Acute ischemia quickly limits oxidative phosphorylation (OXPHOS), and reperfusion does not fully restore energy production when the electron transport chain (ETC) and inner membrane have already been damaged. Notoginsenoside R1 improved ATP levels and several metabolic indices in permanent middle cerebral artery occlusion (MCAO) and OGD models, linking mitochondrial preservation with reduced acute energy failure (Liu et al., 2022). Recovery also requires rebuilding mitochondrial capacity. Orosomucoid 2 (ORM2) promoted neuronal mitochondrial biogenesis through C-C chemokine receptor type 5 (CCR5) and AMP-activated protein kinase (AMPK) signaling in MCAO models, whereas neuron-specific ORM2 loss produced the opposite effect (Jing et al., 2024). Hemorrhagic injury creates a similar energy deficit through different upstream stressors. In hemin-treated primary cortical neurons and collagenase-induced ICH mice, DNA methyltransferase 3 alpha (DNMT3A) increased hypermethylation of the peroxisome proliferator-activated receptor gamma coactivator 1-alpha (PGC-1α) promoter. Pharmacologic or genetic suppression of DNMT3A restored ATP, mitochondrial biogenesis proteins, antioxidant capacity, and behavioral function (Zhao et al., 2026). Mitochondrial one-carbon metabolism provides another layer of metabolic support after ICH. Multi-omics experiments identified methylenetetrahydrofolate dehydrogenase 2 (MTHFD2) as part of a protein kinase R-like endoplasmic reticulum kinase (PERK) and activating transcription factor 4 (ATF4) stress program that helps preserve reducing equivalents and neuronal homeostasis (Liu et al., 2024). In TBI, humanin administered immediately after injury restored oxygen consumption, synaptic proteins, and memory function while reducing inflammatory signaling, showing that bioenergetic rescue can extend to functional plasticity (Thapak et al., 2024). Acute cervical SCI likewise shows persistent depression of mitochondrial respiratory function from 1 to 14 days, while ketone ester treatment partially restores respiratory parameters, particularly at later time points (Seira et al., 2024).

### 2.2 Mitochondrial oxidative stress

Mitochondrial oxidative stress is a major component of secondary CNS injury and reflects sustained reactive oxygen species (ROS) accumulation together with impaired mitochondrial antioxidant control (Fang et al., 2025). In ischemia–reperfusion models, uptake of exogenous functional mitochondria reduced host-cell ROS and improved neuronal and endothelial survival, directly linking mitochondrial state to intracellular oxidative burden (Li et al., 2023). One major source is electron leakage from an impaired ETC, but calcium overload, membrane depolarization, and altered metabolism can amplify the same process. Netrin-1 reduced mtROS and calcium accumulation through protein kinase B (AKT)-dependent regulation of AFG3-like matrix AAA peptidase subunit 2 (AFG3L2). In rats, this intervention also reduced infarct size after cerebral ischemia–reperfusion (Yang et al., 2023). ICH adds oxidative pressure from blood products and iron. Knockdown of NADPH oxidase 4 (NOX4) in rats reduced mtROS, mitochondrial damage, and neuronal pyroptotic signaling, supporting a positive feedback loop between non-mitochondrial and mtROS sources (Ding et al., 2023). TBI produces a comparable redox disturbance. Overexpression of 3-oxoacid CoA-transferase 1 (OXCT1) increased sirtuin 3 (SIRT3), decreased acetylation of superoxide dismutase 2 (SOD2), reduced neuronal ROS, and improved cognitive performance after experimental TBI (Zhuang et al., 2023). More recently, high lactate after TBI was shown to promote SOD2 lactylation through alanyl-tRNA synthetase 2 (AARS2), which reduced SOD2 activity and increased mtROS. SIRT3 activation reversed this modification and decreased neuronal apoptosis in mice (Song J. et al., 2026). Thus, mtROS are not only by-products of injury. They can further damage proteins, membrane lipids, and mtDNA, thereby sustaining metabolic failure and inflammatory signaling.

### 2.3 Mitochondrial dynamics

Mitochondrial dynamics allows mitochondria to reorganize their shape and connectivity during stress, and experimental ischemic models directly link this remodeling to ATP production, mtROS, and neuronal survival (Huang et al., 2024). Dynamin-related protein 1 (DRP1) supports fission, whereas mitofusin 1 (MFN1), mitofusin 2 (MFN2), and optic atrophy 1 (OPA1) support fusion. Direct imaging under ischemic–hypoxic conditions showed that activated DRP1 promotes endoplasmic reticulum-mitochondrial contact formation and excessive fission through actin remodeling, linking structural rearrangement to mitochondrial dysfunction (Duan et al., 2023). Physiologic fission is not inherently harmful because it can isolate damaged mitochondrial regions for later removal. The problem in acute injury is persistent fragmentation that exceeds fusion and clearance capacity. In mouse TBI, mitochondrial fission 1 protein (FIS1)-dependent DRP1 activity increased after injury. Blocking the FIS1-DRP1 interaction for 2 weeks normalized fission-fusion balance and prevented long-term bioenergetic decline, oxidative damage, blood–brain barrier (BBB) deterioration, neurodegeneration, and cognitive impairment (Sridharan et al., 2024). Blast TBI also increased astrocytic DRP1 Ser616 phosphorylation and mitochondrial fragmentation, indicating that mitochondrial dynamics are altered in glia as well as neurons (Guilhaume-Correa et al., 2023). Ischemic neurons show additional regulation through post-translational modification. GCN5-like 1 (GCN5L1) promoted DRP1 acetylation through a cyclin-dependent kinase 5 (CDK5), AMPK, and GCN5L1 axis, increasing fission, mtROS, and apoptosis in OGD and distal MCAO models (Zhang et al., 2024). In SCI, apelin inhibited the mammalian sterile 20-like kinase 1 (MST1), c-Jun N-terminal kinase (JNK), and DRP1 pathway. This increased ATP and ΔΨm while lowering ROS and neuronal apoptosis (Guo Q. et al., 2024).

### 2.4 Mitophagy

Mitophagy provides a selective route for removing dysfunctional mitochondria before they become persistent sources of ROS and mitochondrial danger signals, and enhancing this process can improve mitochondrial integrity after cerebral ischemia–reperfusion (Hou et al., 2025). Recent work on the PTEN-induced kinase 1 (PINK1) pathway showed that ΔΨm controls PINK1 import and retention at the translocase machinery, providing direct evidence for how mitochondrial damage is sensed before Parkin recruitment (Eldeeb et al., 2024). In the canonical pathway, stabilized PINK1 recruits Parkin RBR E3 ubiquitin protein ligase (Parkin) and promotes ubiquitin-dependent recognition of damaged mitochondria. Acute CNS injury can disturb this process at several steps, so increases in individual autophagy proteins do not necessarily indicate complete mitophagic flux. In ICH models and patient samples, PINK1 protein was reduced despite changes in its transcript, while PINK1 overexpression improved ΔΨm, lowered ROS, promoted mitochondrial autophagy, and improved behavioral outcomes (Li et al., 2021). Clearance can also fail downstream. Lipidomics and live-cell imaging in hemin-treated neurons showed that ceramide synthase 6 (CERS6) increased C16 ceramide and interacted with sequestosome 1 (SQSTM1), thereby impairing mitophagy and promoting mitochondrial dysfunction and apoptosis (Xu A. et al., 2025). Ischemic stroke studies provide complementary evidence that restoring mitophagy can be protective. Tetrahydroxy stilbene glucoside enhanced PINK1-Parkin signaling through the ubiquitin-specific peptidase 10 (USP10) and Y-box binding protein 1 (YBX1) axis, increased mitochondria-autophagosome colocalization, and reduced neuronal apoptosis in OGD and MCAO models (Li Y. et al., 2024). SCI can show the opposite pattern. Time-series RNA sequencing from 1 to 7 days after injury indicated suppression of mitophagy during the acute and early subacute period, with excessive ROS and reduced BCL2 interacting protein 3 (BNIP3) contributing to this defect. Enhancing the BNIP3-microtubule-associated protein 1 light chain 3 (LC3) interaction improved mitochondrial clearance and reduced neuronal apoptosis (Yao et al., 2024). In TBI, Parkin overexpression improved mitochondrial quality control and reduced oxidative stress, edema, and behavioral deficits, whereas Parkin loss aggravated injury (Wu et al., 2026).

### 2.5 Mitochondria-derived vesicles

Mitochondria-derived vesicles (MDVs) provide a selective mitochondrial quality-control pathway that can remove damaged mitochondrial proteins and lipids without eliminating the entire organelle. Their formation and cargo composition are stress responsive, and MDVs can function alongside mitophagy to maintain mitochondrial homeostasis (König et al., 2021; Towers et al., 2021; Vasam et al., 2021). Importantly, the fate of MDV cargo may influence inflammatory signaling. OPA1- and SNX9-dependent MDVs can direct selected mitochondrial proteins toward extracellular vesicles, whereas Parkin redirects damaged mitochondrial material toward lysosomes and limits extracellular release of pro-inflammatory mitochondrial DAMPs (Todkar et al., 2021). Because mitochondrial DAMPs can provide upstream inputs to PANoptosis-related signaling, MDV-mediated control of mitochondrial cargo disposal may indirectly modulate the inflammatory environment that favors PANoptotic convergence. However, direct evidence that MDVs regulate integrated PANoptosis or PANoptosome assembly in acute CNS injury is currently lacking. In a neural injury-related model, carbon monoxide preconditioning increased MDV production and improved survival after oxygen–glucose deprivation, although PANoptosis was not directly examined (Guo et al., 2023).

### 2.6 Mitochondrial dysfunction in acute CNS injury

Although ischemic stroke, ICH, SCI, and TBI begin with different primary insults, their secondary injury cascades repeatedly converge on impaired mitochondrial respiration, redox control, dynamics, and quality control (Ong et al., 2025; Thapak et al., 2025). In cerebral ischemia–reperfusion, SIRT3 overexpression restored ΔΨm and ATP synthesis, reduced mtROS, and limited neuroinflammation through the mitochondrial unfolded protein response, providing direct evidence that several mitochondrial defects can be coupled within the same injury process (Xiaowei et al., 2023). Ischemia begins with substrate deprivation and is followed by oxidative and calcium stress during reperfusion. Transplantation of isolated mitochondria in cell and rat MCAO models increased viability, lowered ROS and apoptosis, reduced infarct size, and improved neurological function. Tracking experiments suggested that some mitochondrial components entered host mitochondrial networks while others were processed by lysosomes (Xie et al., 2021). ICH adds heme, iron, thrombin, and perihematomal inflammation. Intravenously delivered astrocytic mitochondria entered the injured brain and neurons in mice, restored SOD2, reduced oxidative injury, and promoted neuroplasticity and functional recovery (Tashiro et al., 2022). SCI combines mechanical disruption with local ischemia and inflammation. Zinc-treated microglia transferred mitochondria to injured neurons through SIRT3 and MFN2 signaling, increasing neuronal ATP and reducing ROS in cell and animal experiments (Guo H. et al., 2024). TBI similarly couples membrane damage and calcium dysregulation with disturbed mitochondrial dynamics and antioxidant defense. Fibronectin type III domain-containing protein 5 (FNDC5) deficiency worsened mitochondrial dysfunction, oxidative stress, apoptosis, and neurological deficits after TBI, whereas SIRT3-dependent restoration of mitochondrial quality control was protective (Ge et al., 2024). Mitochondrial dysfunction in acute CNS injury is actually the result of a combined imbalance in energy production, redox regulation, mitochondrial dynamics, organelle turnover, and mitochondrial signaling. These changes amplify one another and provide an upstream environment for the subsequent convergence of inflammatory signals and multiple regulated cell death pathways.

## 3 PANoptosis and acute CNS injuries

PANoptosis has changed the interpretation of inflammatory cell death because pyroptotic, apoptotic, and necroptotic machinery can be coordinately activated rather than functioning only as isolated programs. The NLR family CARD domain containing 5 (NLRC5) study showed that cellular nicotinamide adenine dinucleotide (NAD^+^) depletion can induce assembly of an NLRC5-containing PANoptosome and activate multiple death pathways (Sundaram et al., 2024). NLR family pyrin domain containing 3 (NLRP3) can also participate directly in PANoptosome formation during prolonged inflammatory stimulation (Sharma et al., 2025). This framework is particularly relevant to acute CNS injury because DAMPs, cytokines, oxidative stress, and organelle injury arise together. In experimental subarachnoid hemorrhage, phosphoglycerate mutase family member 5 (PGAM5) promoted RIPK1 Ser166 phosphorylation and RIPK1-PANoptosome activity, directly demonstrating that a sterile brain insult can engage PANoptotic machinery (Duan et al., 2025). However, evidence strength varies among CNS injuries. The finding that influenza-induced ZBP1-dependent PANoptosis can proceed without the necroptotic executor mixed lineage kinase domain-like protein (MLKL) also illustrates why the presence or absence of a single conventional marker cannot define the entire process (Malireddi et al., 2023).

### 3.1 PANoptosis

PANoptosis is an innate immune inflammatory form of regulated cell death in which pyroptotic, apoptotic, and necroptotic machinery becomes integrated within the same death response. The original PANoptosome study showed that loss of individual pathway components produced limited protection during infection, whereas combined deletion of caspase-1, caspase-11, receptor-interacting serine/threonine-protein kinase 3 (RIPK3), and caspase-8 (CASP8) resulted in much stronger resistance to cell death (Christgen et al., 2020). This functional redundancy is a central feature of PANoptosis because several death modules can compensate when one arm is interrupted. Tumor necrosis factor alpha (TNF-α) and interferon gamma (IFN-γ) provide another example. Their combined stimulation activated gasdermins, apoptotic caspases, and MLKL, while disruption of single pathways was insufficient to fully prevent death (Karki et al., 2021). Cytokine-induced PANoptosis has also been reproduced across multiple human cancer lineages, indicating that the integrated response is not restricted to macrophages (Malireddi et al., 2021). The contribution of each death branch varies with the initiating stimulus and cellular context. Mitochondrial stress can participate upstream. Reverse electron transfer-dependent mtROS promoted oxidation of mtDNA and PANoptosome formation in macrophages. Inhibition of this process simultaneously reduced pyroptotic, apoptotic, and necroptotic signaling.

The contribution of each death branch varies with the initiating stimulus and cellular context. Mitochondrial stress can participate upstream. Reverse electron transfer-dependent mtROS promoted oxidation of mtDNA and PANoptosome formation in macrophages. Inhibition of this process simultaneously reduced pyroptotic, apoptotic, and necroptotic signaling (Shi et al., 2024). Cooperation can also occur among inflammasome sensors. Integrated activation and assembly of NLRP3, absent in melanoma 2 (AIM2), NLR family CARD domain containing 4 (NLRC4), and pyrin drove a coordinated PANoptotic response in experimental systems (Oh et al., 2023). These observations support a flexible cell-death network in which different upstream sensors can converge on caspases, RIPKs, and gasdermins. PANoptosis therefore reflects coordinated pathway plasticity and an integrated response involving several death programs (Figure 2). Throughout this review, “PANoptosis” refers to integrated cell death involving coordinated functional engagement of pyroptotic, apoptotic, and necroptotic machinery. “PANoptotic signaling” denotes coordinated activation of multiple PANoptotic death modules without necessarily implying physical PANoptosome assembly, whereas “PANoptosome-associated signaling” is reserved for settings with biochemical or spatial evidence of cross-pathway complex formation. When such integrated evidence is lacking, we use the broader term “PANoptosis-related signaling” for pathways or molecules implicated in PANoptosis.

**Figure 2:**
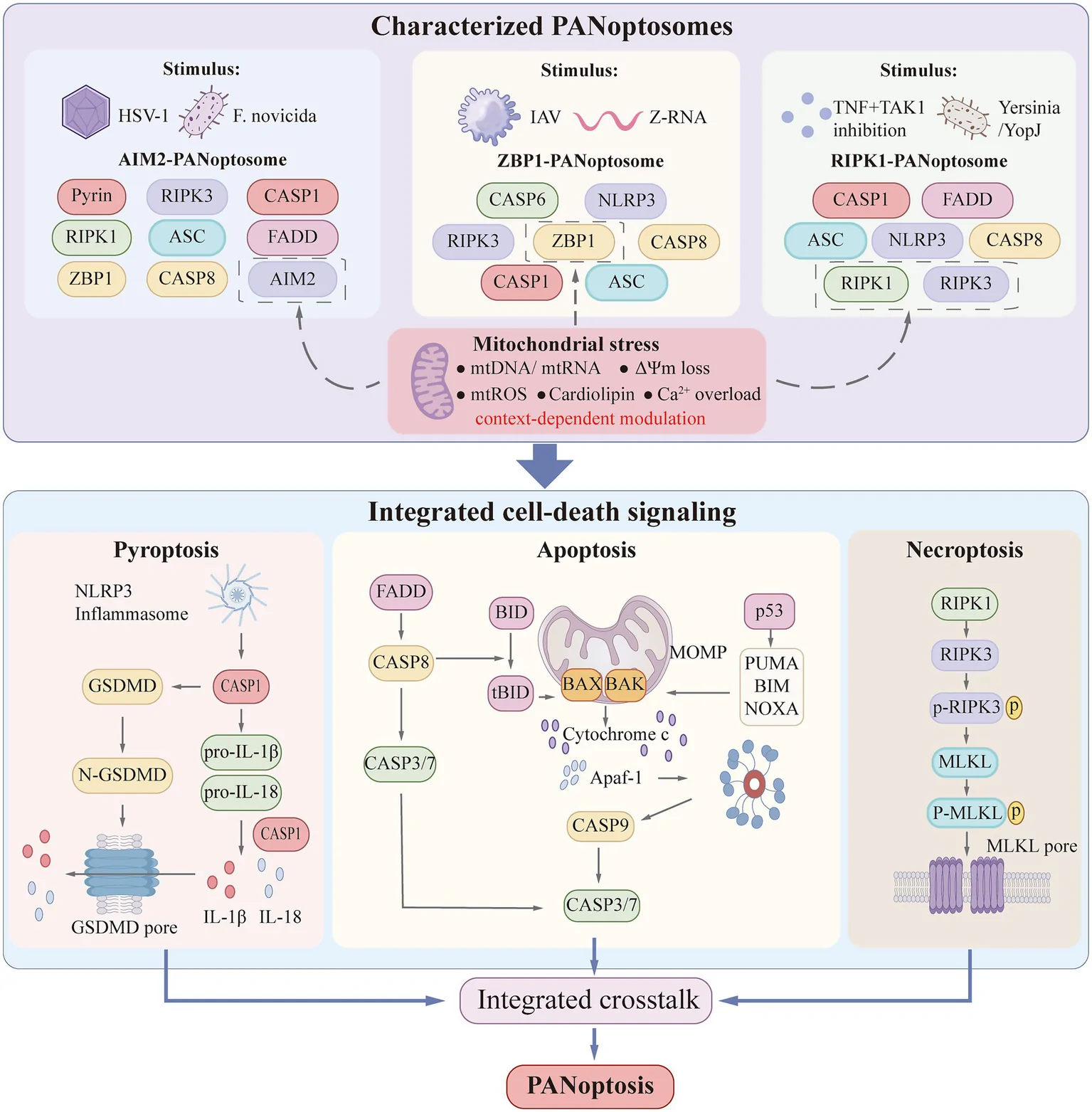
PANoptosis signaling network. Distinct infectious, inflammatory, and stress stimuli can promote the assembly of characterized AIM2-, ZBP1-, or RIPK1-centered PANoptosomes in a stimulus- and context-dependent manner. The NLRP3 inflammasome is shown separately within the pyroptotic module to emphasize that NLRP3 activation alone does not establish PANoptosome formation or PANoptosis. Downstream pyroptotic, apoptotic, and necroptotic pathways involve overlapping components, including CASP1/GSDMD, CASP8 and mitochondrial outer membrane permeabilization (MOMP), and RIPK1/RIPK3/MLKL, respectively. Coordinated engagement of these modules converges through “Integrated crosstalk” before PANoptosis. Mitochondrial stress is presented as a separate modulatory module, including mtROS, mitochondrial nucleic acids, cardiolipin, Ca^2+^ overload, and loss of mitochondrial membrane potential (ΔΨm). Dashed arrows indicate indirect or context-dependent mitochondrial modulation, whereas dashed outlines identify mitochondrial-sensitive components. These connections distinguish potential mitochondrial influences from established stimuli and characterized PANoptosome complexes and do not imply universal direct activation of PANoptosomes by mitochondrial stress.

### 3.2 Panoptosomes

PANoptosomes are dynamic multiprotein complexes that connect upstream sensing with the catalytic and execution machinery of several regulated cell-death pathways. Their composition changes according to the initiating signal and cellular context. The AIM2-PANoptosome contains AIM2, pyrin, ZBP1, apoptosis-associated speck-like protein containing a caspase recruitment domain (ASC), caspase-1, CASP8, Fas-associated protein with a death domain (FADD), RIPK1, and RIPK3. Biochemical interaction experiments demonstrated this complex during *Francisella novicida* and herpes simplex virus 1 infection (Lee et al., 2021). A distinct RIPK1-containing PANoptosome was identified during Yersinia infection, where RIPK1 coordinated inflammasome and apoptotic signaling while also influencing necroptosis (Malireddi et al., 2020). NLR family pyrin domain containing 12 (NLRP12) can form another PANoptosome following combined exposure to heme and pathogen-associated molecular patterns or TNF-α, with CASP8 and RIPK3 acting as important downstream components (Sundaram et al., 2023). PANoptosome assembly is further controlled by regulatory proteins that determine complex stability and downstream signaling. Interferon regulatory factor 1 (IRF1) promotes activation of ZBP1-, AIM2-, RIPK1-, and NLRP12-associated PANoptosomes under different stimuli (Sharma et al., 2023). Protein phosphatase 6 (PP6) favors RIPK1-dependent PANoptosis by shifting RIPK1 phosphorylation toward the pro-death Ser166 state. A clustered regularly interspaced short palindromic repeats (CRISPR) screen showed that loss of its catalytic subunit markedly reduced PANoptotic death induced by transforming growth factor beta-activated kinase 1 (TAK1) inhibition (Bynigeri et al., 2024). Proteins traditionally associated with individual death pathways can also regulate complex assembly. Caspase-6 facilitates ZBP1-RIPK3 interaction and enhances influenza-induced PANoptosis (Zheng et al., 2020). These studies demonstrate the modular nature of PANoptosomes. Identifying the sensor, adaptor, and catalytic modules present in a defined injury context is therefore more informative than assuming a universal complex.

### 3.3 Detection of PANoptosis

At present, no single biomarker can specifically establish PANoptosis, so its identification requires integrated assessment of cell death, activation of the three death branches, and functional crosstalk among them (Tweedell et al., 2024; Li and Qu, 2025). Dedicated experimental workflows therefore first establish that a stimulus actually causes cell death before assigning the underlying mechanism. Real-time live-cell imaging and lactate dehydrogenase (LDH) release assays have been used to quantify the kinetics and magnitude of PANoptotic cell death under different stimuli (Malireddi et al., 2020; Sharma et al., 2023). Calcein acetoxymethyl ester (calcein-AM)/propidium iodide (PI), Hoechst/PI, Annexin V/PI, and terminal deoxynucleotidyl transferase dUTP nick end labeling (TUNEL) can provide complementary information about viability, membrane integrity, and nuclear damage (He et al., 2025). These readouts are not PANoptosis-specific. Staurosporine-treated macrophages, for example, showed early Annexin V positivity and caspase-3/7 activation followed by delayed PI or SYTOX uptake and lytic death, demonstrating why temporal analysis is important (Sarkar et al., 2024). A similar temporal issue was observed after NLRP3 activation, where caspase-1- or gasdermin D (GSDMD)-deficient macrophages were protected from early pyroptosis but developed delayed death associated with activation of additional caspases and RIPKs (Sharma et al., 2025).

After cell death is established, biochemical activation of pyroptotic, apoptotic, and necroptotic machinery should be examined in parallel rather than inferred from total protein abundance alone. Activated caspase-1 together with cleavage of GSDMD or gasdermin E (GSDME), cleavage of CASP8 and executioner caspase-3/7, and phosphorylation of RIPK3 and MLKL provide commonly used biochemical readouts of the three branches (Han et al., 2024; Sundaram et al., 2024). Concurrent activation of GSDMD/GSDME, CASP8, caspase-3/7, and MLKL has also been demonstrated across multiple human cancer cell lines exposed to TNF-α plus IFN-γ (Malireddi et al., 2021). Importantly, these branches are functionally interconnected rather than merely parallel. Direct RIPK3 activation can produce MLKL phosphorylation, caspase activation, and gasdermin cleavage within individual cells, demonstrating reciprocal regulation among the execution pathways (Yang Y. et al., 2026). Extracellular interleukin-1 beta (IL-1β), interleukin-18 (IL-18), high-mobility group box 1 (HMGB1), and LDH can strengthen evidence for inflammatory lysis, but they do not specifically identify PANoptosis. Ninjurin 1 (NINJ1) oligomerization should likewise be interpreted as a readout of terminal plasma membrane rupture because NINJ1 deficiency can reduce membrane rupture and DAMP release without preventing upstream death signaling (Xu Y. et al., 2025). Morphological approaches such as transmission electron microscopy and immunofluorescence can complement these biochemical measurements by showing whether multiple death-associated structural changes coexist in the same experimental setting (Han et al., 2026).

More convincing evidence comes from demonstrating PANoptosome assembly rather than only simultaneous changes in pathway markers. Co-immunoprecipitation can determine whether molecules from different death pathways enter the same complex, while immunoprecipitation-mass spectrometry (IP-MS) can identify additional stimulus-dependent components. The AIM2-PANoptosome study demonstrated interactions among AIM2, pyrin, ZBP1, ASC, caspase-1, CASP8, FADD, RIPK1, and RIPK3 (Lee et al., 2021). NLRP12-associated experiments identified a different sensor-centered complex, supporting the idea that PANoptosome composition is stimulus-dependent rather than fixed (Sundaram et al., 2023). Single-cell spatial evidence can strengthen this conclusion. Expansion microscopy directly visualized colocalization and organization of ASC, CASP8, and RIPK3 in individual PANoptosomes (Wang Y. et al., 2022). Confocal imaging has also shown mitochondrial Z-DNA and oxidized mitochondrial DNA (ox-mtDNA) colocalizing with ZBP1, RIPK3, ASC, and CASP8, while ZBP1 knockdown reduced both complex formation and lytic death (You et al., 2025). Super-resolution imaging and immunoprecipitation have independently demonstrated Z-DNA-ZBP1 colocalization and ZBP1 association with caspase-1, CASP8, and RIPK3 during DNA damage-induced PANoptosis (Yin et al., 2025). Other disease models have similarly identified ZBP1-containing RIPK3-CASP8 complexes, supporting the value of combining spatial imaging with biochemical interaction assays (Zhang X. et al., 2025).

### 3.4 PANoptosis in acute CNS injury

The amount and strength of PANoptosis evidence differ among acute CNS injuries. Integrative analyses of cerebral ischemia–reperfusion and multi-omics studies of SCI already identify coordinated PANoptosis-associated molecular changes, although these signatures remain supportive until cell-death function is demonstrated (Li L. et al., 2026; Xu P. et al., 2026). Evidence in cerebral ischemia has progressed from molecular signatures toward functional intervention and protein interaction. In MCAO and oxygen–glucose deprivation/reoxygenation (OGD/R) models, curcumin-primed olfactory mucosa-derived mesenchymal stem cells reduced AIM2, ZBP1, pyrin, caspase-1, GSDMD, caspase-3, CASP8, RIPK1, RIPK3, and MLKL changes while improving neurological recovery (Lan et al., 2024). More direct evidence came from ZBP1 manipulation. ZBP1 knockdown reduced neuronal death and infarct volume, IP-MS identified RIPK3 as a downstream partner, and RIPK3 knockdown partially reversed injury caused by ZBP1 overexpression (Shu et al., 2025). Melatonin reduced AIM2-associated PANoptotic signaling in penumbral neurons and decreased infarction in rats (Li C. et al., 2026). Vagus nerve stimulation (VNS) also suppressed neuronal PANoptosis through sirtuin 1 (SIRT1; Tang et al., 2025). Single-cell RNA sequencing and spatial transcriptomics further indicate that PANoptosis-associated programs are unevenly distributed among post-stroke cell populations (Bai et al., 2025). Such transcriptional enrichment supports disease association but does not independently establish PANoptotic execution (Jiang et al., 2025).

SCI studies have begun to identify more defined upstream regulators of PANoptotic signaling. Zinc preserved neuronal mitochondrial quality control and attenuated PANoptosis-related signaling through the LGALS3-BAX pathway in oxidative-stress and SCI models (Bai et al., 2024). A separate study combined proteomics, RNA immunoprecipitation sequencing, and IP-MS. YBX1 stabilized Zbp1 messenger RNA and increased ZBP1-mediated neuronal PANoptosis, whereas tripartite motif containing 56 (TRIM56) bound YBX1 and promoted its ubiquitin-dependent degradation (Lou et al., 2024). In spinal cord ischemia–reperfusion injury, the slow-releasing hydrogen sulfide donor GYY4137 reduced apoptotic caspases, NLRP3-GSDMD signaling, and the RIPK1-RIPK3-MLKL axis while improving motor recovery (Xie et al., 2024). Integrated bioinformatic and experimental studies further support extensive crosstalk among regulated death programs after SCI, although such broad signatures are weaker evidence than direct manipulation of ZBP1 or PANoptosome-associated regulators (Tong et al., 2025).

PANoptosis-related evidence in TBI and ICH has emerged more recently, with more direct functional evidence currently available in TBI, whereas evidence in ICH remains largely mechanistic or associative. In TBI, heme and TNF-α cooperatively induced ZBP1-mediated microglial PANoptosis. Ubiquitin-specific peptidase 4 (USP4) interacted with and deubiquitinated ZBP1, while pharmacologic USP4 inhibition with Vialinin A promoted ZBP1 degradation, reduced microglial PANoptosis, and improved functional outcomes (Shuang et al., 2026). This study provides a relatively complete causal chain linking injury-derived stimuli, a defined neural cell type, molecular regulation, and pharmacologic intervention. Evidence in ICH remains less mature. In collagenase-induced ICH mice and BV2 microglia, rhubarb increased small nucleolar RNA 17 (Snord17) and simultaneously reduced ZBP1, RIPK3, NLRP3, and caspase-3, together with inflammation and BBB injury (Lai et al., 2026). Because this evidence primarily relies on pathway markers and functional intervention, it does not yet demonstrate a defined PANoptosome. A five-gene PANoptosis signature has also been associated with immune infiltration and secondary brain injury after ICH, but this remains transcriptomic evidence rather than direct demonstration of PANoptotic death (Chen et al., 2026). Overall, current acute CNS studies increasingly support PANoptotic convergence, but rigorous evidence combining PANoptosome assembly, cell-type localization, and causal perturbation remains concentrated in a limited number of mechanistic studies.

## 4 Mechanistic links between mitochondrial dysfunction and PANoptosis-related signaling in acute CNS injury

Current evidence supports a model in which mitochondria promote PANoptotic convergence through several interconnected danger signals rather than one simple linear pathway. Suppression of mtROS can inhibit ASC, RIPK3, CASP8, and ZBP1-containing PANoptosome formation (Yuan et al., 2024). Opening of the mitochondrial permeability transition pore (mPTP) provides another route, since limiting mPTP-dependent mtDNA release reduces IRF1-ZBP1 signaling and PANoptotic death (Xie et al., 2025). Released mtDNA can also promote AIM2-dependent ZBP1-PANoptosome formation (Guo et al., 2025). Mitochondrial oxidative injury has likewise been placed causally upstream of an AIM2-ZBP1 complex together with pyroptotic, apoptotic, and necroptotic execution (Yang J. et al., 2026). However, the strength of evidence for the complete causal chain varies across different diseases, so direct evidence and mechanistic bridging evidence are discussed separately below. The experimental evidence linking mitochondrial dysfunction with PANoptosis-related signaling across different acute CNS injury models is summarized in Table 1.

**Table 1:** Experimental evidence linking mitochondrial dysfunction with PANoptosis-related signaling in acute CNS injury.

Injury type	Model/cell type	Mitochondrial alteration	PANoptosis-related node	Key experimental evidence	Evidence strength	Ref.
Ischemic stroke	tMCAO rats; OGD/R neurons	Cytosolic oxidized mtDNA accumulation	cGAS-STING	Restricting oxidized mtDNA release reduced neuronal cGAS-STING activation, neuroinflammation, and neurological injury.	Mechanistic bridging evidence	Li Q. et al. (2024)
	MCAO mice; OGD/R neurons	DRP1-dependent fission and mitochondrial fragmentation	ZBP1; NLRP3	Elabela/APJ signaling suppressed ZBP1 while reducing DRP1-dependent fission and NLRP3 activation, with improved neuronal survival and outcome.	Mechanistic bridging evidence	Shen et al. (2024)
	MCAO mice; OGD/R neurons	GSDMD-N-mediated mitochondrial permeabilization with mtDNA and cytochrome c release	IFI204-STING; caspase-1; GSDMD	Neuron-specific IFI204 disruption reduced STING-caspase-1 signaling and mitochondrial permeabilization, interrupting a feed-forward inflammatory injury loop.	Mechanistic bridging evidence	Xu P. et al. (2026)
	MCAO/R mice; OGD/R HT22 cells	Not directly assessed in the PANoptosis study	ZBP1-RIPK3	ZBP1 knockdown reduced neuronal death and infarction; IP-MS identified RIPK3 as a partner, and RIPK3 knockdown partially rescued injury caused by ZBP1 overexpression.	Direct functional PANoptosis evidence	Shu et al. (2025)
Intracerebral hemorrhage	Human perihematomal tissue; collagenase ICH mice; astrocytes	PGAM5-dependent mPTP opening, DRP1 fission, and mtDNA leakage	cGAS-STING	Astrocyte-specific genetic studies linked USP11-PGAM5 to mtDNA escape and cGAS-STING activation; mtDNA depletion or STING blockade reduced neurotoxic astrocyte reactivity and neuronal injury.	Mechanistic bridging evidence	He et al. (2026)
	Collagenase ICH mice; microglia	Mitochondrial DNA-sensing environment; upstream mitochondrial trigger not fully resolved	STING-NLRP3; GSDMD	H151 and microglia-specific Sting deletion reduced NLRP3 activation, GSDMD-mediated pyroptosis, apoptosis, neuroinflammation, and neurological deficits.	Mechanistic bridging evidence	Xue et al. (2025)
	Collagenase ICH mice; BV2 microglia	Not directly established	ZBP1; RIPK3; NLRP3; caspase-3	Rhubarb/Snord17 intervention simultaneously lowered markers assigned to pyroptotic, apoptotic, and necroptotic signaling and improved blood–brain barrier and inflammatory outcomes, but a defined PANoptosome was not demonstrated.	Supportive association	Lai et al. (2026)
Spinal cord injury	Contusion SCI mice; primary microglia	MFN2 loss, disturbed dynamics, and cytosolic mtDNA escape	cGAS-STING	Microglial Mfn2 loss increased cytosolic mtDNA and cGAS-STING signaling; microglia-specific Sting deletion and MFN2-restoring intervention reduced inflammation, neuronal loss, and functional impairment.	Mechanistic bridging evidence	Wei et al. (2024)
	SCI mice; oxidative-stress microglia	SIK2-DRP1 fission, mtROS, and mtDNA leakage	AIM2; caspase-1; GSDMD	SIK2 inhibition reduced mitochondrial fission, mtROS, and mtDNA escape and suppressed AIM2 inflammasome activation and pyroptotic execution.	Mechanistic bridging evidence	Fang et al. (2025)
	SCI mice; neuronal oxidative-stress models	Mitochondrial quality-control failure	LGALS3-BAX; PANoptosis-related execution markers	Zinc preserved neuronal mitochondrial quality control through the LGALS3-BAX axis and reduced multiple PANoptosis-related execution markers and tissue injury.	Mechanistic bridging evidence	Bai et al. (2024)
	SCI mice; neurons; multi-omics and molecular interaction assays	Not directly assessed in the PANoptosis study	YBX1-ZBP1; TRIM56	YBX1 stabilized Zbp1 mRNA and aggravated neuronal PANoptosis; RIP-seq and IP-MS supported the regulatory complex, while TRIM56 promoted YBX1 ubiquitin-dependent degradation.	Direct functional PANoptosis evidence	Lou et al. (2024)
	SCI mice; microglia; bulk/scRNA-seq plus validation	Not directly assessed in the PANoptosis study	IRF1-ZBP1	IRF1 transcriptionally increased ZBP1; pharmacological IRF1 inhibition reduced ZBP1, markers of all three death branches, inflammatory cytokines, and pro-inflammatory microglial polarization.	Mechanistic bridging evidence	Xu X. et al. (2026)
Traumatic brain injury	CCI rats; hippocampal neurons	mPTP-related mtDNA leakage and reduced mitochondrial membrane potential	cGAS-STING-NLRP3	Vagus nerve stimulation restored mitochondrial membrane potential and reduced cytosolic mtDNA; cGAMP weakened protection, whereas cyclosporin A reproduced several protective effects and reduced pyroptotic signaling.	Mechanistic bridging evidence	Ren et al. (2026)
	CCI mice; neuronal models	MFN1-dependent disruption of mitochondrial dynamics and mtROS accumulation	NLRP3; caspase-1; GSDMD	Metformin restored MFN1-dependent mitochondrial homeostasis and suppressed neuronal pyroptosis; Mfn1 silencing markedly weakened the protection.	Mechanistic bridging evidence	Liu T. et al. (2026)
	TBI mice; microglia	Not directly assessed in the PANoptosis study	USP4-ZBP1	Heme and TNF-alpha induced ZBP1-mediated microglial PANoptosis; USP4 stabilized ZBP1, whereas Vialinin A promoted ZBP1 degradation, reduced PANoptosis, and improved functional outcome.	Direct functional PANoptosis evidence	Shuang et al. (2026)

Evidence strength was assigned using the following predefined criteria. Direct functional PANoptosis evidence required demonstration of cell death, activation-specific evidence for pyroptotic, apoptotic, and necroptotic execution, and causal perturbation of a shared PANoptosis regulator that coordinately altered cell death and all three branches. Direct PANoptosome evidence additionally required physical interaction or assembly of components spanning multiple death modules, demonstrated by co-immunoprecipitation, IP-MS, proximity-based assays, or spatial colocalization. Mechanistic bridging evidence indicates causal regulation of one or more PANoptosis-related sensors or execution branches without complete demonstration of integrated PANoptosis. Supportive association indicates concurrent marker, omics, or pathway changes without sufficient functional or complex-level evidence. Changes in total protein abundance, functional outcomes, or multiple death-related markers alone were not considered sufficient to establish direct PANoptosis. AIM2, absent in melanoma 2; cGAS, cyclic GMP-AMP synthase; CCI, controlled cortical impact; DRP1, dynamin-related protein 1; GSDMD, gasdermin D; ICH, intracerebral hemorrhage; IFI204, interferon-activable protein 204; IP-MS, immunoprecipitation-mass spectrometry; MCAO/R, middle cerebral artery occlusion/reperfusion; MFN1/2, mitofusin 1/2; mPTP, mitochondrial permeability transition pore; mtDNA, mitochondrial DNA; mtROS, mitochondrial reactive oxygen species; NLRP3, NLR family pyrin domain containing 3; OGD/R, oxygen–glucose deprivation/reoxygenation; RIPK3, receptor-interacting serine/threonine-protein kinase 3; SCI, spinal cord injury; SIK2, salt-inducible kinase 2; STING, stimulator of interferon genes; TBI, traumatic brain injury; ZBP1, Z-DNA-binding protein 1.

### 4.1 Ischemic stroke

Following ischemia and reperfusion, damaged mitochondria can shift from metabolic casualties to persistent sources of inflammatory death signals. ox-mtDNA accumulates in the neuronal cytosol after transient MCAO and OGD/R, where it activates the cyclic GMP-AMP synthase (cGAS)-stimulator of interferon genes (STING) pathway. Restricting ox-mtDNA release reduces inflammation and neurological injury (Li Q. et al., 2024). STING can subsequently engage the NLRP3 inflammasome. Genetic or pharmacologic STING inhibition reduced microglial caspase-1 activation and GSDMD-dependent pyroptosis in MCAO and OGD/R models (Li W. et al., 2024). A recent study identified an additional feedback mechanism in neurons. Interferon-inducible protein 204 (IFI204) directly interacted with STING and promoted caspase-1-dependent GSDMD cleavage. The GSDMD N-terminal fragment (GSDMD-N) then formed pores in mitochondrial membranes and facilitated the release of mtDNA, cytochrome c, and aconitase 2, which further amplified inflammatory mitochondrial damage (Xu P. et al., 2026). This feed-forward process provides a route through which one mitochondrial lesion can simultaneously strengthen DNA sensing, pyroptotic signaling, and intrinsic apoptotic pressure.

Mitochondrial dynamics and quality control further determine whether these danger signals persist. Elabela signaling through the apelin receptor (APJ) suppressed ZBP1 while reducing DRP1-dependent fission and NLRP3 activation in ischemic neurons, functionally connecting a PANoptotic sensor with mitochondrial fragmentation (Shen et al., 2024). Enhancing mitophagy with terazosin lowered mtROS, NLRP3 activation, and neuronal pyroptosis, while mitophagy inhibition weakened these effects (Wang et al., 2026). In brain microvascular endothelial cells, mitochondrial MLKL oligomerization peaked during early ischemic stress and was inversely associated with later PINK1-Parkin mitophagy (Yang Q. et al., 2026). RIPK3 deficiency also preserved ΔΨm, reduced calcium influx and ROS, and suppressed both necroptotic and apoptotic proteins after ischemic stroke (Wang B. et al., 2022). Lactate-driven bioenergetic failure provides a complementary metabolic link because impaired electron transport promoted RIPK3-dependent endothelial necroptosis after ischemia–reperfusion (Zhou et al., 2026). ROS-thioredoxin-interacting protein (TXNIP)-NLRP3 signaling has also been linked with neuronal necroptotic injury (Liu et al., 2023). Restoration of autophagic flux together with cGAS-STING and NLRP3 inhibition provides another connection between mitochondrial maintenance and inflammatory death (Zhang Y. et al., 2025). These results position ox-mtDNA-STING-NLRP3, ZBP1, and RIPK3 as mitochondrial-sensitive convergence nodes, although a complete mitochondria-to-PANoptosome causal chain remains to be established in ischemic stroke.

### 4.2 Intracerebral hemorrhage

After ICH, one of the strongest mitochondria-to-inflammatory signaling connections involves the ubiquitin-specific peptidase 11 (USP11)-PGAM5 pathway. In human perihematomal tissue, mouse ICH, and astrocyte-specific knockout experiments, PGAM5 promoted mPTP opening and DRP1-dependent mitochondrial fission, which increased cytosolic mtDNA and cGAS-STING activity. Removing mtDNA or blocking STING reduced neurotoxic astrocyte reactivity and secondary neuronal apoptosis (He et al., 2026). A related pathway operates in microglia. Pharmacologic STING inhibition and microglia-specific Sting deletion reduced NLRP3 activation, GSDMD-mediated pyroptosis, apoptosis, inflammatory injury, and neurological deficits after ICH (Xue et al., 2025). Therefore, mitochondrial nucleic acid release after ICH may be an important intermediate step in converting organelle damage into multiple death responses.

### 4.3 Spinal cord injury

SCI provides particularly clear disease-specific evidence for a mitochondria-to-DNA-sensor pathway. Nucleic acids released from damaged mitochondria can transform mitochondrial quality control failure into inflammatory death signals through at least two types of DNA sensing mechanisms. Loss of microglial MFN2 after SCI disrupted mitochondrial dynamics and increased cytosolic mtDNA, which activated cGAS-STING. Microglia-specific Sting deletion reduced inflammatory polarization, neuronal death, and neurological impairment (Wei et al., 2024). A separate study identified salt-inducible kinase 2 (SIK2) as an upstream regulator of DRP1-dependent fission. SIK2 inhibition decreased mtROS and mtDNA leakage and subsequently suppressed AIM2, caspase-1, and GSDMD-N activation in microglia (Fang et al., 2025). DNA sensing can then extend into NLRP3 signaling, since membrane-spanning 4-domains subfamily A member 7 (MS4A7) promoted microglial pyroptosis through the cGAS-STING-NLRP3 axis after SCI (Li et al., 2025a).

Mitophagy determines how long these mitochondrial danger signals persist. Urolithin A increased microglial mitophagy and reduced mtROS, ASC speck formation, caspase-1 activation, and GSDMD-mediated pyroptosis, while 3-methyladenine partially reversed these effects (Chen et al., 2025). Apigenin produced a similar mitophagy-dependent reduction in ROS-NLRP3 signaling, and inhibition of mitophagy weakened its protection (Wu et al., 2025). At the PANoptotic level, integrated multi-omics and experimental validation identified IRF1-driven ZBP1 expression as an important regulator of microglial PANoptosis-related signaling after SCI. IRF1 inhibition reduced ZBP1, several execution markers, inflammatory cytokines, and M1-like microglial polarization (Xu X. et al., 2026). SCI therefore contains strong evidence for a mitochondria-to-DNA-sensor pathway and separate evidence for ZBP1-dependent PANoptosis. Direct demonstration that the same leaked mitochondrial nucleic acids initiate ZBP1-PANoptosome assembly in injured spinal cord remains an important missing step.

### 4.4 Traumatic brain injury

In TBI, mitochondrial membrane damage, disturbed dynamics, and defective mitophagy converge most clearly on mtDNA sensing and NLRP3 signaling. After controlled cortical impact, cytosolic mtDNA increased together with cGAS-STING activity, while genetic loss of cGAS or STING reduced early inflammation and later tissue damage (Fritsch et al., 2022). VNS provides stronger mitochondrial causality. It increased ΔΨm, reduced cytosolic mtDNA, and suppressed cGAS-STING-NLRP3 signaling after TBI. Administration of the STING agonist 2′3’-cyclic GMP-AMP (2′3’-cGAMP) weakened this protection, while the mPTP inhibitor cyclosporin A reproduced several protective effects and reduced caspase-1 and GSDMD-N (Ren et al., 2026). Independent severe-TBI experiments also showed that STING inhibition reduced NLRP3 activation and glial pyroptosis, while STING activation aggravated these responses (Zhang et al., 2022). These data place mitochondrial permeability and cytosolic mtDNA upstream of an important inflammatory execution pathway after TBI.

Mitochondrial dynamics and mitophagy regulate the same inflammatory threshold. Metformin improved mitochondrial homeostasis and reduced NLRP3-dependent neuronal pyroptosis after TBI, but these effects were markedly weakened by MFN1 silencing (Liu T. et al., 2026). Nicotinamide N-oxide enhanced mitophagy, lowered mtROS, and reduced microglial NLRP3, caspase-1, and GSDMD-N, while Mdivi-1 partially reversed its anti-pyroptotic effects (Li et al., 2025b). Astrocyte-derived exosomal gap junction protein alpha 1–20 kDa (GJA1-20 k) also enhanced PINK1-dependent mitophagy and suppressed NLRP3 signaling, with protection reduced in Pink1-deficient mice (Li Y. et al., 2025). TBI can additionally activate RIPK3-containing death complexes. Genetic inhibition of RIPK3 improved outcome after controlled cortical impact, while MLKL deletion did not reproduce the same protection, suggesting that RIPK3 influences inflammatory and death signaling beyond canonical necroptosis (Wu et al., 2021). Together, mtDNA-STING-NLRP3 signaling, defective mitophagy, and RIPK3 activity establish a cellular environment favorable for PANoptotic integration. Direct evidence that mtDNA or mtROS is required for injury-associated ZBP1-PANoptosome assembly in TBI is still lacking. The major cell-type-specific mitochondrial–PANoptosis pathways, biological consequences, and therapeutic implications across experimental injury models are summarized in Table 2.

**Table 2:** Cell-type-specific mitochondrial alterations and PANoptosis-related signaling across acute CNS injury models.

Injury type	Model/cell type	Mitochondrial alteration	PANoptosis-related pathway	Predominant biological function	Evidence strength/therapeutic implication	Ref.
Ischemic stroke	MCAO/R; OGD/R neurons	Oxidized mtDNA accumulation; DRP1-dependent fragmentation; impaired mitochondrial quality control	cGAS-STING/NLRP3 and AIM2; ZBP1-RIPK3	Direct neuronal loss in the ischemic penumbra, infarct expansion, and neurological dysfunction	Direct functional PANoptosis evidence with additional mechanistic bridging evidence; prioritize early mitochondrial stabilization and ZBP1/AIM2 blockade	Li Q. et al. (2024); Li C. et al. (2026); Shen et al. (2024); Shu et al. (2025)
Intracerebral hemorrhage	Human perihematomal tissue; collagenase ICH; neurotoxic astrocytes	USP11-PGAM5-dependent mPTP opening, DRP1 fission, and mtDNA leakage	cGAS-STING; PANoptotic convergence not directly established	Neurotoxic astrocyte reactivity and secondary neuronal apoptosis	Mechanistic bridging evidence; target astrocytic mitochondrial DAMP release/STING while preserving support functions	He et al. (2026)
Intracerebral hemorrhage	Collagenase ICH; BV2/*in vivo* microglia	DNA-sensing/STING-active inflammatory environment; mitochondrial origin of the upstream DNA signal and initiating mitochondrial lesion remain incompletely defined	STING-NLRP3-GSDMD and ZBP1/RIPK3/NLRP3/caspase-3	Cytokine amplification, inflammatory BBB injury, and secondary neuronal damage	Mechanistic bridging evidence and supportive association; target STING or the ZBP1 network, but full PANoptosome validation is needed	Xue et al. (2025); Lai et al. (2026)
Spinal cord injury	Contusion SCI; neurons	Mitochondrial quality-control failure and oxidative stress	LGALS3-BAX; YBX1-ZBP1 with caspase-1/GSDMD, caspase-3, and RIPK3/MLKL	Direct neuronal loss with axonal and circuit disruption	Direct functional PANoptosis evidence with additional mechanistic bridging evidence; preserve mitochondrial quality control and inhibit ZBP1-centered convergence	Bai et al. (2024); Lou et al. (2024)
Spinal cord injury	Contusion SCI; pro-inflammatory microglia	MFN2 loss and mtDNA escape; SIK2-DRP1 fission/mtROS; low AMPK activity	cGAS-STING/AIM2 plus IRF1-ZBP1 PANoptosis	Sustained cytokine production and inflammatory amplification with secondary neuronal injury	Direct functional PANoptosis evidence with additional mechanistic bridging evidence; AMPK activation or IRF1/ZBP1 inhibition may be useful in inflammatory microglia	Wei et al. (2024); Fang et al. (2025); Liu S. et al. (2026); Xu X. et al. (2026)
Traumatic brain injury	CCI/stretch; cortical and hippocampal neurons	Defective PINK1 mitophagy, ROS accumulation, and disturbed MFN1-dependent homeostasis	Mitochondrial quality-control failure permits coordinated PANoptotic execution; NLRP3 is an additional convergence node	Direct neuronal loss and long-term cognitive/motor dysfunction	Direct functional PANoptosis evidence with additional mechanistic bridging evidence; enhance PINK1 mitophagy and protect mitochondria early	Xu L. et al. (2025); Liu T. et al. (2026)
Traumatic brain injury	CCI; heme/TNF-α-exposed microglia	Direct mitochondrial trigger not established; injury DAMP-rich environment	USP4-ZBP1 PANoptosis; context-dependent PANoptotic signaling	Usually inflammatory amplification and cognitive injury, but elimination of highly activated microglia can reduce net inflammation	Direct functional PANoptosis evidence; avoid blanket inhibition or induction and target defined microglial states	Zheng et al. (2025); Shuang et al. (2026)
Ischemic stroke	OGD/R brain microvascular endothelial cells	ETC dysfunction; mitochondrial MLKL oligomerization; insufficient PINK1-Parkin mitophagy	RIPK3-MLKL necroptotic branch; full PANoptosome not established	BBB and microvascular failure with secondary inflammatory exposure	Mechanistic bridging evidence; preserve endothelial bioenergetics/mitophagy before assigning PANoptosis	Yang Q. et al. (2026); Zhou et al. (2026)
Subarachnoid hemorrhage	Endovascular perforation rats; neurons	Cytosolic accumulation of mitochondrial PGAM5	PGAM5-RIPK1 Ser166 phosphorylation; RIPK1-PANoptosome (ASC/RIPK3/caspase-1/caspase-8)	Direct neuronal PANoptosis and early brain injury	Direct PANoptosome evidence; useful acute-CNS benchmark, with PGAM5/RIPK1 as candidate targets	Duan et al. (2025)

Evidence strength follows the predefined criteria used in Table 1. The biological function column refers to the dominant tissue-level consequence of death or stress signaling in the specified cell population and should not be interpreted as an exclusive function. Direct cell-type-resolved evidence for mitochondria-associated PANoptosis in oligodendrocytes/oligodendrocyte precursor cells remains insufficient. AIM2, absent in melanoma 2; BBB, blood–brain barrier; CCI, controlled cortical impact; cGAS, cyclic GMP-AMP synthase; DRP1, dynamin-related protein 1; ETC, electron transport chain; GSDMD, gasdermin D; ICH, intracerebral hemorrhage; MFN1/2, mitofusin 1/2; mPTP, mitochondrial permeability transition pore; mtDNA, mitochondrial DNA; mtROS, mitochondrial reactive oxygen species; NLRP3, NLR family pyrin domain containing 3; OGD/R, oxygen–glucose deprivation/reoxygenation; PINK1, PTEN-induced kinase 1; RIPK1/3, receptor-interacting serine/threonine-protein kinase 1/3; SAH, subarachnoid hemorrhage; SCI, spinal cord injury; STING, stimulator of interferon genes; TBI, traumatic brain injury; ZBP1, Z-DNA-binding protein 1.

## 5 Therapeutic strategies targeting mitochondrial dysfunction and PANoptosis-related signaling in acute CNS injuries

Because mitochondrial dysfunction supplies several upstream signals for PANoptosis, mitochondria-directed therapy may suppress a common source of death signaling before pyroptotic, apoptotic, and necroptotic execution becomes fully established. In SCI models, preservation of mitochondrial quality control has been associated with reduced PANoptosis-related signaling and neuronal injury (Bai et al., 2024). The strongest evidence comes from interventions that improve mitochondrial function and simultaneously suppress PANoptosis or convergence nodes such as NLRP3, AIM2, ZBP1, RIPK3, or cGAS-STING. Many studies, however, still examine only one execution branch. Such interventions are better described as approaches that may restrain PANoptotic convergence unless coordinated pathway suppression or PANoptosome-related regulation is directly demonstrated. This distinction is important because mitochondrial rescue may be biologically relevant to PANoptosis even when a study was originally designed to investigate only pyroptosis, apoptosis, or necroptosis. The principal therapeutic strategies targeting mitochondrial dysfunction and PANoptosis-related signaling are summarized in Table 3.

**Table 3:** Therapeutic strategies targeting mitochondrial dysfunction and PANoptosis-related signaling.

Injury type	Intervention	Primary mitochondrial/PANoptotic target	Mechanistic effect	Experimental evidence	Limitations	Ref.
Ischemic stroke	SIRT3 overexpression	SIRT3; mitochondrial unfolded protein response	Restores membrane potential and ATP synthesis, lowers mtROS, and restrains neuroinflammatory stress.	MCAO/R and OGD/R models showed improved mitochondrial function and reduced cerebral injury.	Gene delivery and rapid target engagement remain challenging in acute stroke, and the clinically relevant treatment window is undefined. PANoptosis was not directly evaluated.	Xiaowei et al. (2023)
	Naotaifang formula	DRP1/FIS1; OPA1/MFN1/MFN2	Rebalances fission and fusion by suppressing excessive DRP1-dependent fission and promoting fusion.	MCAO/R mice and OGD/R cells; DRP1 overexpression weakened protection and DRP1 silencing reproduced key benefits.	Active constituents, batch consistency, pharmacokinetics, and target specificity remain incompletely defined. The efficacy of delayed treatment and direct effects on PANoptosis require further validation.	She et al. (2025)
	USP7 overexpression	USP7-SIRT1-PINK1/Parkin mitophagy	Stabilizes SIRT1 and restores PINK1/Parkin-dependent clearance of damaged mitochondria.	Cerebral ischemia–reperfusion models; autophagy inhibition or SIRT1/PINK1 knockdown abolished much of the protection.	Gene-based manipulation limits immediate clinical translation, while broad USP7 deubiquitinase activity may cause off-target effects. Its therapeutic window and direct effects on PANoptosis remain unclear.	Hou et al. (2025)
	Melatonin	AIM2-associated PANoptotic signaling	Reduces AIM2 expression and coordinated PANoptosis-related execution in penumbral neurons.	Rat cerebral ischemia/reperfusion with multimodal MRI and histology; reduced infarction and PANoptotic protein changes.	Evidence remains preclinical, and optimal dose and post-ischemic treatment timing require clarification. Mitochondrial dependence and direct PANoptosome regulation have not been demonstrated.	Li C. et al. (2026)
	Mitochondrial transplantation	Mitochondrial bioenergetics and redox homeostasis	Supplies functional mitochondrial components, improves respiration, and reduces ROS and apoptosis.	Cell and rat MCAO models; improved viability, infarct volume, and neurological function with tracking of donor mitochondrial components.	Donor mitochondrial quality, isolation and delivery methods, immunogenicity, persistence, and scalability require standardization. Whether transplanted mitochondria achieve durable functional integration with host networks remains uncertain.	Xie et al. (2021)
Intracerebral hemorrhage	Celastrol	OPA1-dependent mitochondrial fusion	Restores mitochondrial morphology and fusion, lowers oxidative stress, and reduces neuronal apoptosis.	ICH mice and primary neurons; neuron-specific Opa1 deletion largely abolished protection.	Its pleiotropic pharmacology complicates target attribution, while dose, brain exposure, systemic safety, and the therapeutic window remain uncertain. PANoptosis was not directly evaluated.	Diao et al. (2024)
	MSC-derived exosomes	OPA1-mediated mitochondrial fusion	Promotes neuronal mitochondrial fusion and respiratory recovery, reducing oxidative stress and apoptosis.	ICH mice and neuronal models; neuron-specific Opa1 deletion blunted exosome-mediated mitochondrial and functional recovery.	Donor and cargo heterogeneity, manufacturing reproducibility, biodistribution, dosing, and target-cell uptake remain major barriers. Direct suppression of PANoptosis has not been established.	Xu Y. et al. (2026)
	MTK458	PINK1-Parkin mitophagy	Enhances mitophagic clearance and suppresses NLRP3/caspase-1/GSDMD-dependent pyroptosis.	Autologous-blood ICH mice showed less edema, improved behavior, increased mitophagy, and reduced inflammatory pyroptosis.	Preventive administration in part of the experimental design limits clinical relevance. Delayed-treatment efficacy and long-term safety require validation, and coordinated PANoptosis was not directly demonstrated.	Li J. et al. (2025)
	H151	STING	Blocks STING-dependent NLRP3 activation, pyroptosis, apoptosis, and neuroinflammation.	Collagenase ICH mice; pharmacologic STING inhibition and microglia-specific Sting deletion produced convergent protection.	The optimal therapeutic window remains undefined, and prolonged STING inhibition may compromise innate immune defense. Full suppression of coordinated PANoptosis has not yet been demonstrated.	Xue et al. (2025)
	Angiopep-2-modified extracellular vesicles carrying Pgam5 siRNA	USP11-PGAM5-mPTP-DRP1-mtDNA-cGAS-STING axis	Reduces mitochondrial permeability/fission and mtDNA escape, thereby limiting DNA-sensing inflammatory signaling.	ICH mouse studies used targeted delivery and astrocyte-focused pathway validation; neurological outcomes improved.	Targeted nanodelivery requires further validation of manufacturing, biodistribution, cellular uptake, scalability, and repeat-dose safety. Clinically realistic post-injury treatment timing also remains uncertain.	He et al. (2026)
Spinal cord injury	SS-31 (elamipretide)	Mitochondrial cardiolipin and oxidative phosphorylation	Preserves mitochondrial membrane integrity and bioenergetics while reducing oxidative stress and neuronal injury.	*In vitro* and in vivo SCI models showed mitochondrial protection and improved neurobiological outcomes.	Optimal dose, CNS exposure, treatment duration, and clinically relevant therapeutic window remain uncertain. Whether mitochondrial protection suppresses coordinated PANoptosis rather than individual death pathways is unresolved.	Ravenscraft et al. (2025)
	MFN2-restoring biomimetic MASM7 delivery	MFN2-mtDNA-cGAS-STING	Restores mitochondrial dynamics, limits mtDNA escape, and suppresses microglial DNA-sensing inflammation.	SCI mice; intervention complemented Mfn2/Sting genetic experiments and improved functional recovery.	Target-cell delivery, biodistribution, nanoparticle scalability, and long-term safety require further validation. Sustained MFN2 manipulation may also affect physiological mitochondrial dynamics.	Wei et al. (2024)
	Zinc	LGALS3-BAX-dependent mitochondrial quality control	Preserves mitochondrial integrity and attenuates PANoptosis-related signaling in neurons.	Oxidative-stress cell models and SCI animals showed improved mitochondrial quality control and reduced PANoptosis-related signaling/markers and neurological injury.	Zinc has a narrow homeostatic range and broad systemic effects, making dose optimization and cell-specific targeting difficult. The clinically effective treatment window also remains unclear.	Bai et al. (2024)
	IRF1 inhibition	IRF1-ZBP1	Reduces ZBP1 expression and downstream pyroptotic, apoptotic, and necroptotic execution in microglia.	Multi-omics-guided SCI experiments with pharmacological inhibition reduced PANoptosis markers, cytokines, and inflammatory polarization.	IRF1 has broad immune and transcriptional functions, so sustained inhibition may impair protective immune responses. Cell-specific delivery, treatment timing, and upstream mitochondrial causality remain unresolved.	Xu X. et al. (2026)
	Zinc-enhanced mitochondrial transfer	SIRT3-MFN2-dependent microglia-to-neuron mitochondrial transfer	Increases neuronal ATP, decreases ROS, and restores damaged mitochondrial networks.	Cell and animal SCI experiments showed mitochondrial transfer and improved motor recovery.	Transfer efficiency, recipient-cell specificity, persistence, and the fate of transferred mitochondria remain difficult to control. Durable functional integration and the effects of zinc pleiotropy also require clarification.	Guo H. et al. (2024)
Traumatic brain injury	OXCT1 overexpression	OXCT1-SIRT3-SOD2 antioxidant axis	Increases SIRT3, reduces SOD2 acetylation and ROS, and limits neuronal death.	Mouse TBI with AAV-mediated neuronal OXCT1 overexpression improved cognitive outcomes and oxidative injury.	AAV-mediated overexpression may not achieve sufficiently rapid expression for acute TBI. Cell specificity, systemic metabolic effects, treatment timing, and direct PANoptosis regulation remain uncertain	Zhuang et al. (2023)
	Metformin	AMPK-MFN1-dependent mitochondrial dynamics	Restores mitochondrial homeostasis, lowers mtROS, and suppresses NLRP3-dependent neuronal pyroptosis.	CCI and cellular models; MFN1 knockdown substantially weakened metformin-mediated protection.	Dose, CNS exposure, injury-severity dependence, and the optimal post-injury window require clarification. Current evidence mainly supports mitochondrial and pyroptotic regulation rather than direct PANoptosis inhibition.	Liu L. et al. (2026)
	Vagus nerve stimulation	mPTP-mtDNA-cGAS-STING-NLRP3 axis	Improves mitochondrial membrane potential, limits cytosolic mtDNA, and suppresses inflammatory pyroptosis.	CCI rats; cGAMP antagonized and cyclosporin A mimicked key protective effects, supporting pathway causality.	Stimulation intensity, duration, timing, and patient selection require optimization, while invasive implementation may limit acute use. Full inhibition of PANoptotic signaling has not been demonstrated.	Ren et al. (2026)
	C-176	STING	Suppresses acute STING/type-I interferon neuroinflammation and reduces secondary tissue injury.	CCI mice treated 30 min after injury showed smaller lesions, improved gait parameters, and reduced inflammatory cytokine expression.	Treatment was initiated very early after injury, limiting clinical generalizability. Long-term STING inhibition may interfere with host defense, and mitochondrial dependence and PANoptosis were not directly tested.	Fryer et al. (2024)
	Vialinin A	USP4-ZBP1	Inhibits USP4-dependent ZBP1 stabilization, promotes ZBP1 degradation, and suppresses microglial PANoptosis.	TBI mouse and microglial experiments linked heme/TNF-alpha to ZBP1 PANoptosis and showed functional protection after USP4 inhibition.	USP4 has multiple substrates, raising potential off-target and immune effects. Brain pharmacokinetics, cell-specific delivery, delayed-treatment efficacy, and upstream mitochondrial dependence remain to be established.	Shuang et al. (2026)

Many interventions were evaluated using preventive or very early post-injury treatment, and their efficacy after clinically relevant delayed administration remains uncertain. Broad or sustained inhibition of PANoptosis-related signaling may also interfere with host defense, clearance of irreversibly damaged cells, or subsequent tissue remodeling. AIM2, absent in melanoma 2; AMPK, AMP-activated protein kinase; BBB, blood–brain barrier; cGAS, cyclic GMP-AMP synthase; CCI, controlled cortical impact; DRP1, dynamin-related protein 1; ICH, intracerebral hemorrhage; IRF1, interferon regulatory factor 1; MFN1/2, mitofusin 1/2; mPTP, mitochondrial permeability transition pore; mtDNA, mitochondrial DNA; mtROS, mitochondrial reactive oxygen species; NLRP3, NLR family pyrin domain containing 3; OPA1, optic atrophy 1; PINK1, PTEN-induced kinase 1; SCI, spinal cord injury; SIRT3, sirtuin 3; SOD2, superoxide dismutase 2; STING, stimulator of interferon genes; TBI, traumatic brain injury; UPRmt, mitochondrial unfolded protein response; USP4/7, ubiquitin-specific peptidase 4/7; VNS, vagus nerve stimulation; ZBP1, Z-DNA-binding protein 1.

### 5.1 Targeting mitochondrial oxidative stress

Targeting mitochondrial oxidative stress is attractive because mtROS can simultaneously impair respiratory proteins, oxidize mtDNA, disturb calcium handling, and amplify inflammatory death signaling. In SCI, the mitochondria-targeted peptide elamipretide (SS-31) restored ΔΨm, lowered mtROS, inhibited NLRP3 inflammasome activation and pyroptosis, and improved locomotor recovery (Jiang et al., 2023). A newer contusion study found that SS-31 also preserved OXPHOS proteins, reduced BAX and cleaved caspase-3, and supported long-term neuronal and synaptic remodeling (Song Z. et al., 2026). These findings indicate that sustained mitochondrial protection can reduce both inflammatory and apoptotic pressure rather than simply lowering a bulk ROS signal.

The same principle is supported in acute brain injury. In cerebral ischemia–reperfusion, SIRT3 overexpression reduced mtROS, preserved ΔΨm and ATP synthesis, and limited neuroinflammation through the mitochondrial unfolded protein response (UPRmt; Xiaowei et al., 2023). Netrin-1 reduced mtROS and calcium accumulation through AKT-dependent regulation of AFG3L2 and decreased infarct size in rats (Yang et al., 2023). In TBI, increasing OXCT1 enhanced the SIRT3-SOD2 antioxidant axis, reduced neuronal ROS and apoptosis, and improved cognition (Zhuang et al., 2023). In ICH, celastrol reduced mitochondrial oxidative stress and neuronal apoptosis through OPA1-dependent mitochondrial fusion (Diao et al., 2024).

### 5.2 Modulating mitochondrial dynamics

Therapeutic control of mitochondrial dynamics should restore fission-fusion balance rather than abolish fission because physiologic division remains necessary for segregation and clearance of damaged mitochondrial segments. In cerebral ischemia–reperfusion, Naotaifang reduced DRP1 and FIS1 while restoring OPA1, MFN1, and MFN2. DRP1 overexpression weakened its protection, whereas DRP1 silencing improved mitochondrial morphology and neuronal survival (She et al., 2025). Astragaloside IV combined with ligustrazine similarly corrected abnormal DRP1 SUMOylation and reduced excessive fission after cerebral ischemia–reperfusion (Chen et al., 2024). These intervention experiments are important because they move beyond correlations between mitochondrial fragmentation and tissue damage and show that manipulation of the fission machinery changes outcome.

Fusion-directed approaches are also promising. After ICH, mesenchymal stem cell-derived exosomes restored OPA1-dependent fusion, mitochondrial respiration, and neuronal viability, while neuron-specific OPA1 deletion largely abolished these benefits (Xu Y. et al., 2026). In SCI, apelin suppressed the MST1-JNK-DRP1 pathway, increased ATP and ΔΨm, and reduced ROS and neuronal apoptosis (Guo Q. et al., 2024). In TBI, metformin improved MFN1-dependent mitochondrial homeostasis and reduced NLRP3 activation and neuronal pyroptosis, while MFN1 knockdown weakened protection (Liu T. et al., 2026). Pharmacologic disruption of the FIS1-DRP1 interaction during the acute period after TBI also prevented chronic bioenergetic decline, oxidative injury, BBB deterioration, and cognitive deficits (Sridharan et al., 2024). Restoring dynamics may therefore shorten the persistence of fragmented, ROS-producing mitochondria and indirectly raise the threshold required for PANoptotic convergence.

### 5.3 Targeting mitophagy

Mitophagy offers a different therapeutic advantage because it removes organelles that continuously generate mtROS and release mitochondrial DAMPs. In ICH mice, the PINK1 activator MTK458 enhanced PINK1-Parkin mitophagy and simultaneously reduced NLRP3 activation, caspase-1 signaling, inflammatory cytokines, cortical pyroptosis, edema, and neurological deficits (Li J. et al., 2025). In cerebral ischemia–reperfusion, ubiquitin-specific peptidase 7 (USP7) stabilized SIRT1 and promoted PINK1-Parkin mitophagy. Its protection was lost after autophagy inhibition or SIRT1/PINK1 knockdown, showing that mitophagic flux was functionally required (Hou et al., 2025). Ginsenoside Rg1 also enhanced PINK1-Parkin-dependent mitophagy while reducing mtROS and microglial NLRP3 activation (Sui et al., 2024).

Microglial mitophagy is particularly relevant to SCI and TBI because damaged mitochondria in immune cells can sustain local inflammatory signaling. It may attenuate PANoptotic amplification by simultaneously reducing the number of damaged mitochondria and decreasing the release of DAMPs. In SCI, inhibition of the P2Y6 receptor (P2Y6R) with MRS2578 activated PINK1-Parkin mitophagy, shifted microglia toward a reparative phenotype, and improved functional recovery (Lin et al., 2025). Triggering receptor expressed on myeloid cells 1 (TREM1) inhibition with LP17 similarly activated AMPKα-PINK1-Parkin signaling and reduced microglial inflammation (Xia et al., 2026). Zinc enhanced PINK1-Parkin mitophagy, stabilized mitochondrial membrane control through BAX, BCL2 antagonist/killer 1 (BAK), and voltage-dependent anion channel 1 (VDAC1), reduced mtDNA leakage, and suppressed cGAS-STING signaling after SCI (Jin et al., 2026). In TBI, astrocyte-derived extracellular vesicles enriched in GJA1-20 k activated PINK1-dependent mitophagy and suppressed NLRP3 signaling, with weaker protection in Pink1-deficient mice (Li Y. et al., 2025).

### 5.4 Promoting mitochondrial biogenesis

Mitophagy removes damaged organelles, but recovery also requires their replacement with respiration-competent mitochondria. Biogenesis therapy simultaneously replenishes ATP production capacity and reduces the metabolic load borne by individual damaged mitochondria. Biogenesis therapy therefore focuses mainly on AMPK-PGC-1α signaling and its downstream effectors nuclear respiratory factor 1 (NRF1) and mitochondrial transcription factor A (TFAM). In ICH mice, the G protein-coupled receptor 39 (GPR39) agonist TC-G 1008 activated cAMP response element-binding protein (CREB)-PGC-1α signaling, increased NRF1, TFAM, mtDNA copy number, and antioxidant enzymes, and reduced neuronal degeneration (Zhang Z. et al., 2025). In cerebral ischemia–reperfusion, the PGC-1α agonist ZLN005 increased mitochondrial number and uncoupling protein 2 (UCP2), reduced mitochondrial oxidative stress, and limited neuronal injury (Yang et al., 2025). DL-3-n-butylphthalide promoted neuronal mitochondrial biogenesis through AMPK-PGC-1α signaling after ischemia–reperfusion (Li and Zhu, 2025).

This approach also extends to traumatic CNS injury. Photobiomodulation after SCI activated AMPK-PGC-1α-TFAM signaling, restored respiratory-chain activity and ATP production, and reduced neuronal apoptosis, while AMPK inhibition attenuated these effects (Zhu et al., 2022). A melatonin-containing brain extracellular matrix hydrogel further enhanced neural stem-cell mitochondrial metabolism and neuroregeneration through the AMPK-PGC-1α-NRF1/TFAM axis after SCI (Wei et al., 2026). In TBI, ZLN005 restored OXPHOS gene expression, mitochondrial structure, and respiratory function in inflammatory neuronal models and improved cognition in mice (Dong et al., 2025). Biogenesis alone is unlikely to restrain PANoptosis if damaged mitochondria continue to accumulate. Combining biogenesis with mitophagy may be more effective because it couples removal of DAMP-producing organelles with restoration of metabolic reserve.

### 5.5 Targeting mitochondrial DAMP sensing and PANoptosis-related signaling

Blocking the transmission of mitochondrial DAMPs to innate immune sensors may reduce upstream signaling that contributes to PANoptotic convergence. ox-mtDNA is an actionable target because its cytosolic accumulation links mitochondrial injury to DNA-sensing inflammatory pathways. In cerebral ischemia–reperfusion, limiting cytosolic release of ox-mtDNA reduced neuronal cGAS-STING activation, inflammation, and neurological injury (Li Q. et al., 2024). In SCI, restoration of MFN2 with a biomimetic MASM7 delivery system reduced mtDNA escape, suppressed microglial cGAS-STING activity, and improved functional recovery (Wei et al., 2024). In ICH, the BBB-permeable STING antagonist H-151 reduced NLRP3 activation, GSDMD-mediated microglial pyroptosis, apoptosis, and motor deficits (Xue et al., 2025). These results demonstrate that reducing mitochondrial danger signals can simultaneously weaken multiple inflammatory death inputs.

Greater specificity may be achieved by targeting defined PANoptosis regulators or, where complex-level evidence is available, PANoptosome components. Melatonin reduced AIM2-associated PANoptotic signaling and infarction in the ischemic penumbra (Li S. et al., 2026). ZBP1 knockdown reduced ischemic neuronal PANoptosis and infarct volume, while RIPK3 knockdown partially reversed injury caused by ZBP1 overexpression, supporting the ZBP1-RIPK3 axis as a therapeutic node (Shu et al., 2025). In SCI, pharmacologic inhibition of IRF1 decreased ZBP1 and several PANoptotic execution markers in microglia (Xu X. et al., 2026). In TBI, Vialinin A inhibited USP4-dependent stabilization of ZBP1, promoted ZBP1 degradation, reduced microglial PANoptosis, and improved functional outcome (Shuang et al., 2026). These studies demonstrate synergistic changes in death mechanisms. Future strategies may require the combined inhibition of mitochondrial DAMP release and disease-specific PANoptosome receptors to reduce cell death at both the upstream and complex levels.

### 5.6 Transfer and transplantation

Mitochondrial transfer and transplantation differ from the preceding strategies because they replace or supplement dysfunctional organelles rather than only modifying endogenous signaling. In cerebral ischemia–reperfusion, transplantation of isolated mitochondria increased oxidative respiration and cell viability, reduced ROS and apoptosis, decreased infarct volume, and improved neurological outcomes. Tracking showed that some donor mitochondrial components entered host mitochondrial networks, while others were processed by lysosomes (Xie et al., 2021). After ICH, intravenously delivered astrocytic mitochondria entered injured brain tissue and neurons, restored SOD2, reduced oxidative injury, enhanced neurite growth, and improved recovery (Tashiro et al., 2022). Magnetically responsive artificial cells further improved mitochondrial delivery to ICH lesions and shifted microglia toward a less inflammatory state (Zhou et al., 2025). Therefore, the quality of donor mitochondria, transport efficiency, and the selection of recipient cells may collectively determine the therapeutic efficacy.

Intercellular mitochondrial transfer can also be therapeutically enhanced. In SCI, zinc promoted SIRT3-MFN2-dependent transfer of mitochondria from microglia to injured neurons, increasing neuronal ATP, reducing ROS, and improving motor recovery (Guo H. et al., 2024). Photobiomodulation increased connexin 36 (Cx36)-dependent neuronal uptake of transplanted mitochondria and strengthened ATP restoration, antioxidant capacity, tissue repair, and locomotor recovery in rats (Zhu et al., 2023). Direct transplantation has also been tested after TBI. Exogenous mitochondria were internalized by neurons, astrocytes, and microglia, reduced neuronal apoptosis, increased astrocytic brain-derived neurotrophic factor (BDNF), and improved spatial memory and anxiety-related behavior (Zhao et al., 2021). To date, strong metabolic and functional evidence for acute CNS injury has been established; however, it has not yet been proven that mitochondrial transplantation can directly prevent PANoptosome assembly. Its anti-PANoptosis potential is primarily based on restoring ATP, reducing mtROS, and limiting the release of mtDNA and other mitochondrial DAMPs.

## 6 Limitations and prospects

Despite growing evidence linking mitochondrial dysfunction to PANoptosis in acute CNS injury, the field remains limited by how PANoptosis is defined experimentally. Many studies still infer PANoptosis from concurrent changes in pyroptotic, apoptotic, and necroptotic markers, while direct demonstration of PANoptosome assembly, pathway interdependence, and rescue by genetic perturbation remains uncommon. Standardized criteria that combine cell-death kinetics, cleavage or phosphorylation of execution proteins, complex formation, and functional intervention are therefore needed (Tweedell et al., 2024). This is particularly important in injured neural tissue, where neurons, microglia, astrocytes, endothelial cells, and infiltrating immune cells may activate different death programs at different times (Tang et al., 2026). Differences in injury severity, reperfusion status, animal age, sex, and sampling time can further change both mitochondrial responses and the apparent contribution of each death pathway (Gao et al., 2026).

A second limitation is that the proposed mechanistic link between mitochondrial dysfunction and PANoptosis is often reconstructed from separate experiments rather than demonstrated as one continuous causal sequence. mtROS, ox-mtDNA, mitochondrial permeability, disturbed dynamics, and defective mitophagy can each activate inflammatory or death-related pathways, but their relative importance is likely to vary with injury type, cell identity, and disease stage (Pan et al., 2026; Ye et al., 2026). Future studies should combine live-cell mitochondrial reporters with single-cell or spatial transcriptomics, proteomics, proximity-based interaction assays, and cell-type-specific genetic models. Such approaches could determine when mitochondrial damage becomes irreversible, which mitochondrial DAMPs initiate specific PANoptosomes, and whether the dominant PANoptotic sensor changes from the acute to the subacute phase (Goel et al., 2026).

Translation presents an additional challenge. Most evidence comes from rodent models or cultured cells, while human validation, pharmacokinetic data, and clinically relevant therapeutic windows remain limited (Zhang et al., 2026). Importantly, several interventions were administered prophylactically or very shortly after injury, whereas patients with acute CNS injury often present after variable delays. Future studies should therefore establish efficacy under delayed-treatment paradigms. Broad or sustained suppression of PANoptosis-related signaling may also impair host defense, clearance of irreversibly damaged cells, or reparative tissue remodeling, supporting temporal and cell-selective modulation rather than persistent pathway blockade (Gao et al., 2026). Mitochondrial transfer and transplantation introduce further questions concerning donor source, isolation quality, storage, delivery route, cellular uptake, persistence, and long-term safety (Meng et al., 2025; Riou et al., 2025). Future work should therefore prioritize human-derived organoids or induced pluripotent stem cell models, prospective biospecimen studies, and standardized outcome measures (Shima et al., 2025). Ultimately, the most useful therapeutic strategy may not be maximal inhibition of a single death pathway. A more realistic goal is to identify mitochondrial convergence points that can suppress pathological PANoptotic amplification while preserving adaptive mitochondrial quality control and necessary immune responses (Fang et al., 2026).

## 7 Conclusion

Acute CNS injuries initiated by ischemia, hemorrhage, or mechanical trauma rapidly develop into complex secondary injury cascades. Across ischemic stroke, ICH, SCI, and TBI, mitochondrial dysfunction is consistently associated with metabolic failure, oxidative stress, inflammatory signaling, and regulated cell death, and in some experimental settings can contribute upstream to these processes. Impaired OXPHOS, excessive mtROS, disturbed fission and fusion, defective mitophagy, altered MDV trafficking, and insufficient mitochondrial renewal can progressively destabilize cellular homeostasis. These changes may create conditions in which pyroptotic, apoptotic, and necroptotic machinery becomes coordinately engaged through PANoptosis-related signaling. This convergence helps explain why neuronal loss, glial activation, and tissue inflammation often evolve together after the initial injury. Together, these observations support a potential mechanistic convergence between mitochondrial dysfunction and PANoptosis-related signaling. Damaged mitochondria can release mtDNA and other DAMPs, increase oxidative pressure, and disturb calcium and metabolic homeostasis. These signals can engage cGAS-STING, NLRP3, AIM2, ZBP1, RIPK1, RIPK3, caspases, and other components that are also involved in PANoptosis-related signaling and downstream cell death. At the same time, excessive mitochondrial fission and insufficient mitophagy can prolong the persistence of damaged mitochondria, which further amplifies inflammatory and death signals. Current evidence is consistent with potential bidirectional crosstalk, whereby mitochondrial injury may facilitate PANoptosis-related signaling, while inflammatory cell death can further aggravate mitochondrial dysfunction; however, a continuous causal relationship has not been demonstrated in most acute CNS injury models. Accordingly, direct evidence linking mitochondrial dysfunction to integrated PANoptosis or defined PANoptosome assembly remains limited, whereas much of the current literature is better interpreted as mechanistic bridging evidence or supportive association. The dominant mitochondrial signals, PANoptosome-related sensors, and responding cell populations are unlikely to be identical across different CNS injuries. Their relative contribution may also shift from the acute phase to later stages of secondary injury. This integrated view also broadens the therapeutic landscape. Strategies that reduce mitochondrial oxidative stress, restore mitochondrial dynamics, enhance mitophagy, promote mitochondrial biogenesis, limit mitochondrial DAMP signaling, or provide functional mitochondria through intercellular transfer or transplantation may simultaneously attenuate several mitochondrial and inflammatory inputs that contribute to PANoptosis-related signaling. Where causal links have been experimentally established, targeting mitochondrial DAMP-sensing pathways or defined PANoptosis regulators may provide additional specificity by interrupting the transition from organelle stress to coordinated cell-death signaling. Combining mitochondrial repair with selective control of validated PANoptosis-related pathways could potentially provide broader protection than targeting a single downstream execution mechanism. The therapeutic goal should therefore not be indiscriminate suppression of cell death or innate immunity, but rather preservation of mitochondrial quality together with selective restraint of pathological PANoptosis-related signaling. Defining the cell-specific, temporal, and causal relationships between mitochondrial dysfunction and PANoptosis will be essential for determining when mitochondria-associated PANoptotic signaling represents a valid therapeutic target in acute CNS injury.
